# FuTURe Study (FUnctional Tricuspid Update Study of REcurrence)—Personalized Risk Stratification for Functional Tricuspid Regurgitation Recurrence After Mitral–Tricuspid Surgery

**DOI:** 10.3390/jcm15166152

**Published:** 2026-08-07

**Authors:** Maria Grandinetti, Gabriele Mazzenga, Piergiorgio Bruno, Giovanni Alfonso Chiariello, Annalisa Pasquini, Maria Calabrese, Nicola Testa, Marialisa Nesta, Monica Filice, Rosa Lillo, Federico Cammertoni, Natalia Pavone, Francesco Burzotta, Massimo Massetti

**Affiliations:** 1 Department of Cardiovascular Sciences, Fondazione Policlinico Universitario A. Gemelli IRCCS, 00168 Rome, Italy; maria.grandinetti@policlinicogemelli.it (M.G.); gabriele.mazzenga@guest.policlinicogemelli.it (G.M.); giovannialfonso.chiariello@policlinicogemelli.it (G.A.C.); annalisa.pasquini@policlinicogemelli.it (A.P.); maria.calabrese@policlinicogemelli.it (M.C.); nicola.testa@policlinicogemelli.it (N.T.); marialisa.nesta@policlinicogemelli.it (M.N.); monica.filice@policlinicogemelli.it (M.F.); rosa.lillo@policlinicogemelli.it (R.L.); federico.cammertoni@policlinicogemelli.it (F.C.); natalia.pavone@policlinicogemelli.it (N.P.); francesco.burzotta@policlinicogemelli.it (F.B.); massimo.massetti@policlinicogemelli.it (M.M.); 2 Faculty of Medicine and Surgery, Catholic University of the Sacred Heart, 00168 Rome, Italy

**Keywords:** tricuspid valve, tricuspid surgery, functional tricuspid regurgitation, personalized medicine, risk stratification

## Abstract

**Objectives**: Functional tricuspid regurgitation (TR) recurrence after concomitant mitral–tricuspid surgery remains associated with adverse long-term outcomes, while reintervention on the isolated tricuspid valve carries substantial operative risk. Despite increasing awareness of the prognostic importance of functional TR, concomitant tricuspid valve repair remains inconsistently adopted in contemporary practice, particularly in technically demanding procedures. We sought to identify predictors of recurrent TR and develop an individualized prediction model to support patient-tailored surgical planning. **Methods**: This single-center ambispective study included 178 consecutive patients undergoing concomitant mitral–tricuspid surgery between January 2012 and June 2019. Baseline clinical, echocardiographic and operative variables were retrospectively collected, whereas long-term clinical and echocardiographic follow-up was prospectively completed after Ethics Committee approval. Clinical, echocardiographic and operative variables were first evaluated by univariable Cox proportional hazards analysis. Based on their univariable association with recurrent TR, biological plausibility and clinical relevance, candidate predictors were subsequently entered into a multivariable Cox proportional hazards model while limiting model complexity according to the number of available outcome events. Internal validation was performed by bootstrap resampling, and regression coefficients were transformed into an exploratory individualized perioperative nomogram estimating 5-year tricuspid regurgitation recurrence-free survival. **Results**: Univariable analysis identified female sex, dyslipidemia, left ventricular ejection fraction < 45%, severe pre-operative TR, indexed tricuspid annular diameter and suture-based annuloplasty as predictors of recurrent TR. Multivariable analysis identified surgical repair strategy as the only independent predictor associated with recurrent TR, with suture-based annuloplasty showing a significantly higher recurrence risk than prosthetic ring annuloplasty. These variables were integrated into an exploratory individualized prediction model estimating 5-year recurrence-free survival: the FuTURe nomogram. **Conclusions**: Our findings confirm the association between prosthetic ring annuloplasty and a lower risk of recurrent TR, consistent with current evidence supporting ring implantation as the preferred repair strategy. Beyond identifying predictors of recurrence, the FuTURe study proposes an exploratory perioperative individualized prediction model intended to complement current guideline recommendations. Consistent with its acronym, the FuTURe study shifts the focus from immediate procedural success to the patient’s future clinical trajectory, highlighting the value of individualized recurrence-risk assessment from a lifetime management perspective.

## 1. Introduction

In approximately 90% of cases, tricuspid regurgitation (TR) is secondary, typically resulting from pressure and/or volume overload leading to right ventricular dilatation or enlargement of the right atrium and tricuspid annulus. Most cases of secondary TR are associated with left-sided valvular disease [1]. Historically, functional tricuspid regurgitation (FTR) was frequently overlooked because correction of the left-sided valve lesion was believed to halt or reverse TR progression. Over the last four decades, however, accumulating evidence has demonstrated that untreated FTR often persists or progresses despite successful left-sided surgery, with a detrimental impact on survival and clinical outcomes [2,3,4]. This paradigm shift has renewed interest in tricuspid valve (TV) disease and recently led to the development of the first Tricuspid Valve Academic Research Consortium (TVARC) consensus document [5]. Current evidence strongly supports concomitant tricuspid valve repair when indicated, with ring annuloplasty generally considered the most durable repair strategy because of its superior annular stability and lower recurrence rates compared with suture techniques [6,7]. Accordingly, contemporary guidelines increasingly advocate a proactive approach to FTR. Nevertheless, despite these recommendations, significant variability in surgical practice persists. Concomitant tricuspid repair is still frequently omitted or simplified, particularly in technically demanding settings such as minimally invasive mitral surgery, reflecting the additional operative complexity, longer cardiopulmonary bypass times, and higher costs, and the learning curve associated with prosthetic ring implantation [7]. While current guidelines effectively identify patients who should undergo concomitant tricuspid intervention, they provide limited support for individualized estimation of post-operative recurrence risk. Consequently, surgical planning still relies largely on surgeon experience and local practice patterns rather than objective prediction of long-term outcomes. In this setting, individualized risk stratification may complement guideline recommendations by supporting patient-tailored decision-making and encouraging treatment of the tricuspid valve in patients who might otherwise remain untreated. Despite the renewed focus on tricuspid surgery, recurrent TR after mitral–tricuspid procedures remains an important clinical challenge, occurring at an estimated rate of 1.9% per year [8]. Because isolated tricuspid valve reintervention carries substantial operative risk, identifying patients at increased risk of recurrence during the index operation has become increasingly relevant. The identification of prognostic factors has become increasingly relevant across different fields of valve surgery, where individualized risk stratification may help optimize long-term outcomes [9]. Furthermore, the expanding role of transcatheter tricuspid therapies underscores the importance of accurate risk prediction to optimize both the initial surgical strategy and long-term follow-up. The present study sought to investigate the clinical, echocardiographic and intraoperative determinants of recurrent TR and to develop an individualized prediction model estimating recurrence risk. Rather than challenging current guideline recommendations, our aim was to explore whether individualized recurrence-risk estimation could complement them by supporting a more tailored surgical strategy once the indication for concomitant tricuspid repair has already been established.

## 2. Materials and Methods

### 2.1. Study Design and Patient Population

The FuTURe Study (FUnctional Tricuspid Update study of Recurrence) is a single-center ambispective study involving 178 patients who underwent combined mitral and tricuspid valve surgery at our institution from January 2012 to June 2019. Patients were screened for TV repair during mitral surgery for degenerative mitral disease. The exclusion criteria included combined procedures (e.g., aortic surgery or myocardial revascularization), endocarditis, cardiac reoperation, pregnancy, and breastfeeding. The study, approved by our Local Ethics Committee (ID 6050), adhered to the 1976 Declaration of Helsinki (and amendments) and STROBE guidelines [10]. Written informed consent was obtained from all participants. Baseline demographic, clinical, echocardiographic, operative and perioperative data were retrospectively collected from institutional records. Following Ethics Committee approval in 2023, long-term follow-up was prospectively conducted through a dedicated institutional follow-up program.

Degenerative mitral valve disease included leaflet prolapse, flail leaflet, fibroelastic deficiency and Barlow’s disease. During the study period, the institutional indications for concomitant tricuspid repair remained substantially unchanged and reflected both contemporary guideline recommendations and our institutional philosophy of proactively treating annular dilatation to minimize the risk of undertreatment. Indications included severe functional TR or tricuspid annular dilatation (>40 mm or >21 mm/m^2^ indexed), even in the presence of mild-to-moderate regurgitation.

**Figure 1:**
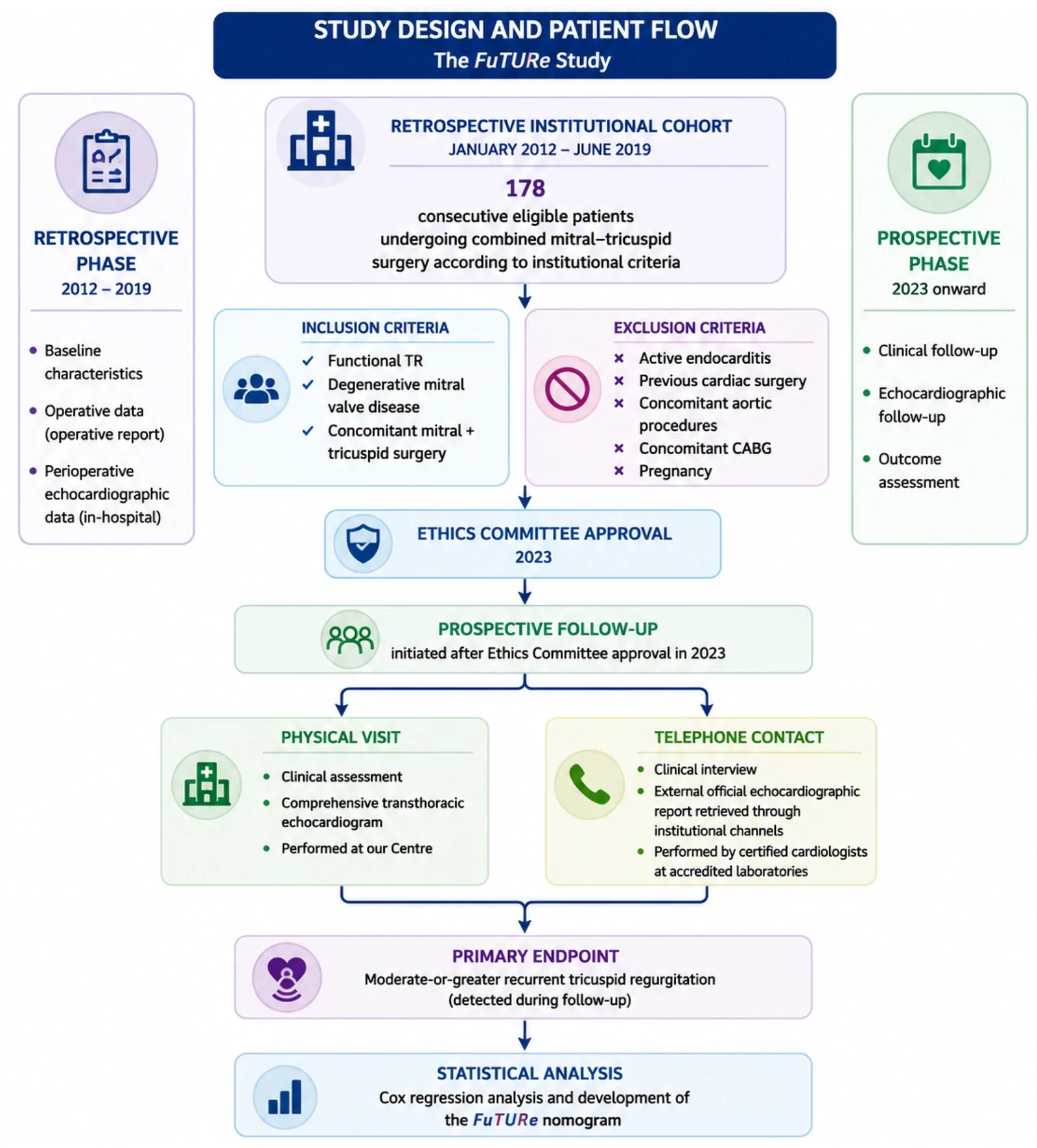
Flow diagram illustrating patient selection, retrospective data collection, prospective follow-up, endpoint adjudication and final analytical cohort.

The primary objective of the study was to identify independent predictors of long-term recurrent TR following concomitant mitral–tricuspid surgery using pre-operative clinical, echocardiographic, and intraoperative variables. The secondary objective was to develop an exploratory individualized prediction model estimating 5-year recurrence-free survival: the FuTURe nomogram. The overall study design, including patient selection, retrospective data collection, prospective follow-up and endpoint assessment, is summarized in Figure 1.

### 2.2. Echocardiography

Pre-operative comprehensive echocardiography is routinely performed and reported in our Cardiac Surgery Echolab by highly trained operators. Chamber quantification, left ventricle (LV) ejection fraction (EF) and diastolic function were assessed according to the most recent guidelines; comprehensive assessment of right ventricle (RV) geometry and systolic function was performed according to current recommendations [10,11]. RV disfunction was defined as the presence of at least one of TAPSE (Tricuspid Annular Plane Systolic Excursion) < 16 mm, RV FAC (Fractional Area Change) < 35% or an S’ (Tissue Doppler-derived tricuspid lateral annular systolic velocity) < 10 cm/s. The severity of TR was graded according to a multi-parametric approach, including assessment of VC (vena contracta) width, EROA (Effective Regurgitant Orifice Area), regurgitant volume, regurgitant fraction and hepatic vein systolic flow reversal [11,12]. Right atrial (RA) enlargement was defined as the presence of at least one of RA area > 18 cm^2^ (moderate > 26 cm^2^, severe > 32 cm^2^), RA major dimension > 53 mm (moderate > 57 mm, severe > 63 mm) or RA minor dimension > 44 mm (moderate > 48 mm, severe > 53 mm) [11].

### 2.3. Operative Technique

TV repair with a ring was performed according to the standard technique [13]. Ringless TV repair was performed according to the De Vega technique [14] or the previously described De-Kay technique [15,16]. In selected patients, suture tricuspid annuloplasty was performed after aortic cross-clamp removal on the beating heart or during cardioplegia administration, according to surgeon preference and operative conditions. The choice between prosthetic ring annuloplasty and suture-based annuloplasty (De Vega or De-Kay) was left to the operating surgeon according to patient characteristics, intraoperative findings and surgical judgment, reflecting routine clinical practice throughout the study period. For the statistical analyses, De Vega and De-Kay annuloplasty were analyzed as a single suture-based annuloplasty group. Although technically distinct, both procedures share the same principle of ringless annular reduction. Because De Kay annuloplasty represented only a small minority of ringless repairs, separate analyses were considered underpowered to provide reliable estimates and were therefore not pursued. In addition, the present study was not designed to compare different suture-based repair techniques, but rather prosthetic ring versus ringless annuloplasty.

### 2.4. Data Collection

Patient data were retrieved from the institution’s electronic archives. Long-term follow-up was initiated after Ethics Committee approval in 2023 and consisted primarily of dedicated outpatient clinical and echocardiographic reassessment at our institutional Heart Valve Clinic [17]. Whenever attendance was not feasible, patients underwent structured telephone interviews, and the most recent official echocardiographic examination performed by certified cardiologists at accredited laboratories was retrieved through institutional channels. External echocardiographic evaluations represented only a minority of follow-up examinations, and all reports were independently reviewed and validated by the investigators before endpoint adjudication. Complete clinical and echocardiographic follow-up was achieved in all 178 patients included in the final analytical cohort. Median follow-up was 6 years (IQR 3–7), estimated using the reverse Kaplan–Meier method.

### 2.5. Outcome Definition

The primary objective of the study was to identify predictors of recurrent moderate-or-greater functional tricuspid regurgitation during long-term follow-up. All patients underwent routine transthoracic echocardiography before hospital discharge. Moderate-or-greater tricuspid regurgitation detected at this examination was classified as residual TR and was not considered part of the primary endpoint. Patients presenting with residual TR remained under longitudinal follow-up and were considered to have reached the study endpoint only if further progression occurred after hospital discharge. Recurrent TR was defined as moderate-or-greater tricuspid regurgitation identified after hospital discharge following an initially satisfactory repair. The date of recurrence corresponded to the first echocardiographic examination demonstrating recurrent TR.

### 2.6. Sample Size

Few studies have examined predictive factors for TR recurrence, and no individualized prediction model has been proposed to estimate patient-specific recurrence risk. Using our institutional database, data from 178 patients treated between January 2012 and June 2019 were analyzed. Following van Smeden’s approach, which allows for events per variable (EPV) below the traditional threshold of EPV ≥ 10 [18], and assuming a 15% event rate and 10 covariates, the sample size supported scenarios with an R^2^ of 0.2 and SD of 1.0, under a 95% bilateral confidence interval. Study power was calculated using Cox regression in PASS2022 software, with a log Hazard Ratio (B) of 0.60 while holding other covariates constant.

### 2.7. Statistical Analysis

Descriptive statistics summarized clinical and demographic data. Qualitative variables were expressed as frequencies, while quantitative variables were presented as means ± SD or medians with IQR, as appropriate. The Shapiro–Wilk test assessed Gaussian distribution. Missing data were imputed using the imputeR 2.2 package: Lasso regression for quantitative data and classification trees for qualitative data. Differences between surgical techniques were tested with the Fisher–Freeman–Halton exact test or Chi-squared test for qualitative variables, and Student’s *t*-test or the Mann–Whitney U test for quantitative variables. Potential predictors of recurrent TR were evaluated using Cox proportional hazards regression models reporting Hazard Ratios (HRs) with 95% confidence intervals (CIs). The proportional hazards assumption was assessed by graphical inspection of hazard functions and Schoenfeld residuals. No relevant violations of proportionality were observed; therefore, the final multivariable model and the FuTURe nomogram were derived using a standard Cox proportional hazards regression model [19,20]. Kaplan–Meier analysis evaluated differences in TR recurrence-free survival (RFS) between techniques, using the log-rank test and cumulative incidence functions. The R packages “ggplot2” and “survminer” were applied. Predictors for the multivariable model were selected considering their biological plausibility, previously reported prognostic relevance, clinical judgment and univariable association with recurrent TR, while deliberately limiting model complexity according to the number of available outcome events, in line with van Smeden [18] and TRIPOD recommendations [21]. The multivariable model provided β coefficients and SEs, and performance was assessed using indices like C-index, Somers’ Dxy, Nagelkerke R2, calibration intercept/slope, and Brier score [22]. The Hosmer–Lemeshow test assessed goodness-of-fit. The c-index (AUC) quantified model accuracy, and corrected Dxy measured model optimism. The “Rms 6.7-0”, “magrittr 2.0.3”, and “predtools 0.0.3” packages were used. The scoring system assigned integer values based on β coefficients, plotted into a nomogram for outcome prediction. Total scores were mapped to the survival axis. Internal validation used bootstrap resampling (200 repetitions) and calibration plots, illustrating predicted vs. observed outcomes. Statistical significance was set at *p* < 0.05, with suggestive values (0.05 ≤ *p* < 0.10) also reported. All analyses were performed with R software v4.3.0.

## 3. Results

### 3.1. General Characteristics of the Study Sample

Overall, we included in the study 178 patients who received tricuspid surgery (both the De Vega and De-Kay ringless repair techniques and ring repair) due to FTR during mitral valve surgery. Table 1 reports clinical characteristics of the study population and the comparison between the ring group (n = 54) and De Vega/De-Kay group (n = 124; 102 De Vega and 22 De-Kay).

### 3.2. Surgical, Post-Operative and Follow-Up Data

**Figure 2:**
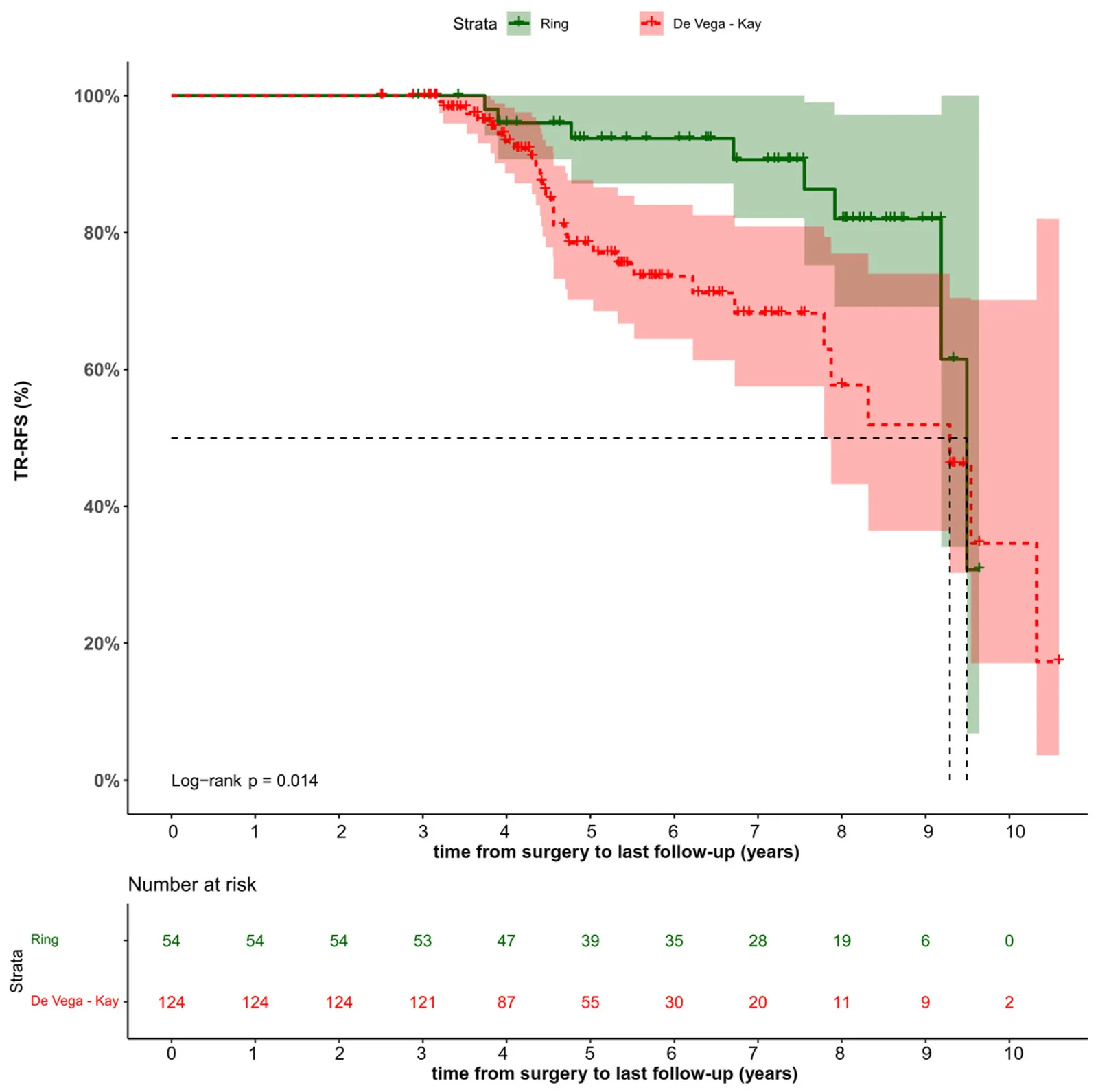
Kaplan–Meier survival curves for Tricuspid Regurgitation Recurrence-Free Survival (TR-RFS) on the entire sample (N = 178), stratified by surgical technique, either De Vega/De-Kay (red line) or ring repair (green line).

All patients underwent surgery for TR, with the De Vega/De-Kay technique predominating over ring repair (69.7% vs. 30.3%). Surgical details are summarized in Table 2. Complete clinical and echocardiographic follow-up was available for all 178 patients included in the study. Median follow-up was 6 years (IQR 3–7), estimated using the reverse Kaplan–Meier method. No deaths were identified during the available investigator-ascertained long-term follow-up. However, 6 patients (3.4%) required repeat cardiac surgery (e.g., for endocarditis), and 45 (25.3%) were hospitalized, nearly half for cardiovascular issues (e.g., PTCA). Post-operative AV block (mostly transient) occurred in 24% of cases with no significant difference between the ring and no-ring groups. Moderate to severe recurrent TR was observed in 21.3% of cases (N = 38), with a higher prevalence following De Vega/De-Kay surgery (24.2% vs. 14.8%; log-rank *p* = 0.014) (Figure 2). The median time to recurrent TR was 69.7 months (IQR 48.1–85.8), significantly shorter in the De Vega/De-Kay group (*p* < 0.001) (Table 2).

### 3.3. Residual and Recurrent TR

Before hospital discharge, all patients underwent routine transthoracic echocardiographic evaluation. Moderate residual TR was identified in nine patients (eight treated with suture-based annuloplasty and one with prosthetic ring annuloplasty). None of these patients experienced further progression during follow-up; therefore, residual TR was not considered a recurrent event and was excluded from the primary endpoint analysis.

### 3.4. Potential Predictors of Recurrence-Free Survival (RFS)

We investigated the association between baseline clinical, echocardiographic and operative variables and long-term tricuspid regurgitation recurrence-free survival (TR-RFS). Univariable analysis identified female sex (HR 2.09; *p* = 0.045), dyslipidemia (HR 1.95; *p* = 0.050), left ventricular ejection fraction < 45% (HR 3.26; *p* = 0.002), indexed tricuspid annular diameter (HR 1.13; *p* = 0.033) and suture-based annuloplasty (HR 2.62; *p* = 0.018) as significant predictors, while severe pre-operative TR and longer aortic cross-clamp time showed suggestive associations (see Table 3).

### 3.5. Development of the FuTURe Predictive Model

**Figure 3:**
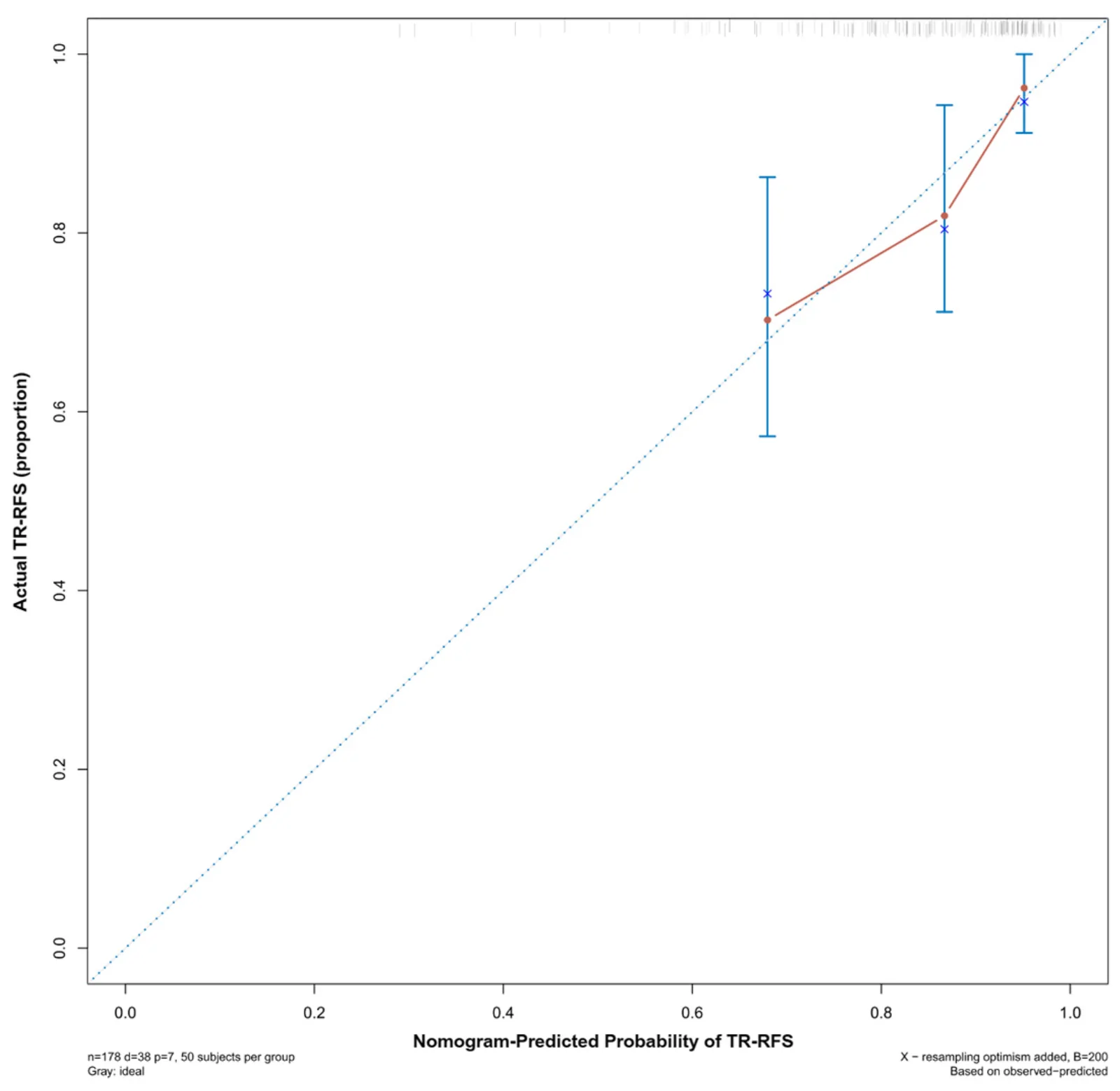
Calibration plot obtained after bootstrap internal validation demonstrating satisfactory agreement between predicted and observed recurrence-free survival (abbreviations: TR- RFS: tricuspid regurgitation recurrence-free survival).

**Figure 4:**
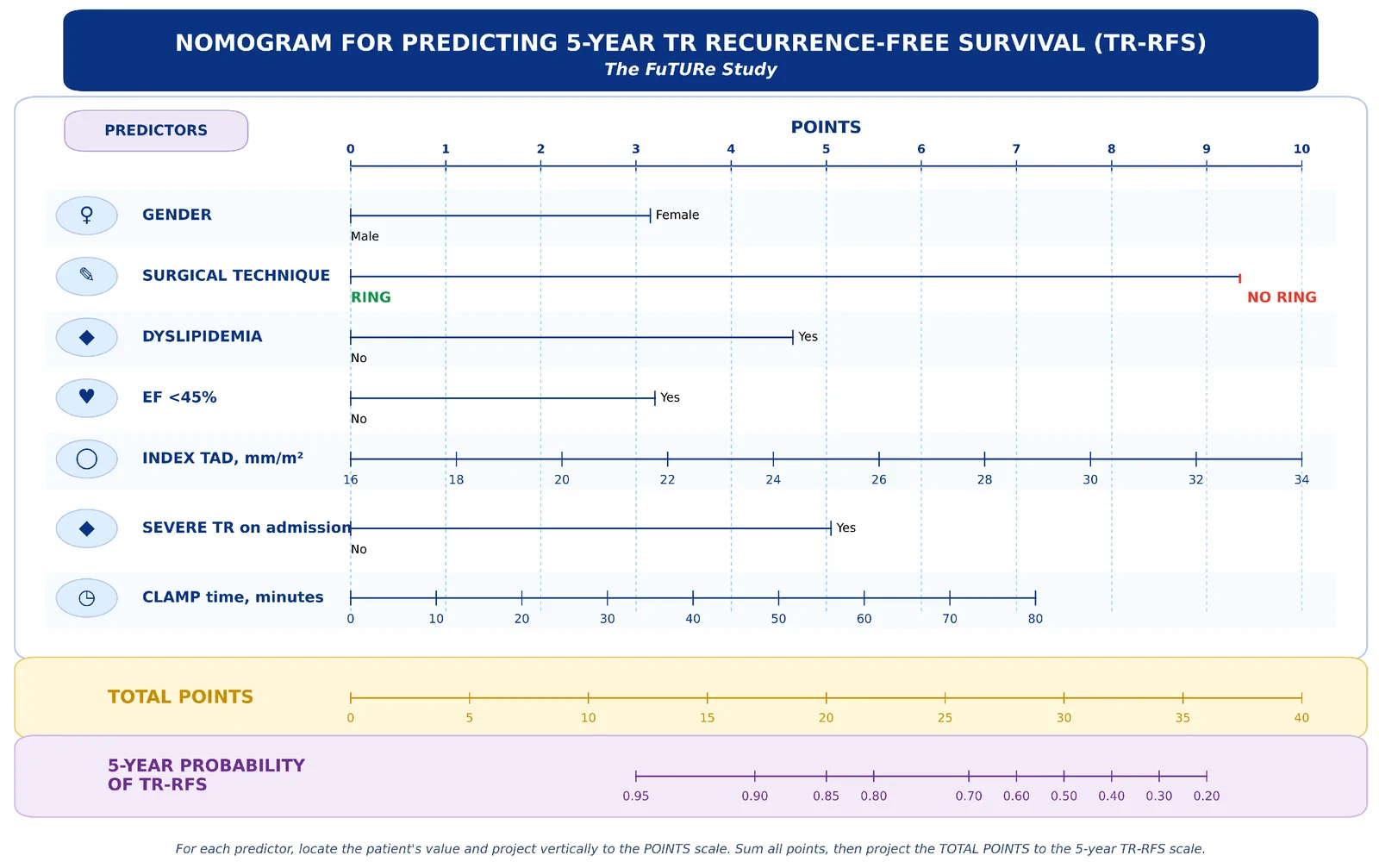
FuTURe nomogram. Exploratory individualized perioperative prediction model estimating the probability of 5-year tricuspid regurgitation recurrence-free survival according to baseline clinical, echocardiographic and operative characteristics. (Abbreviations: TR: Tricuspid Regurgitation; EF: Ejection Fraction; TAD: Tricuspid Annular Diameter; TR-RFS: Tricuspid Regurgitation Recurrence-Free Survival).

**Figure 5:**
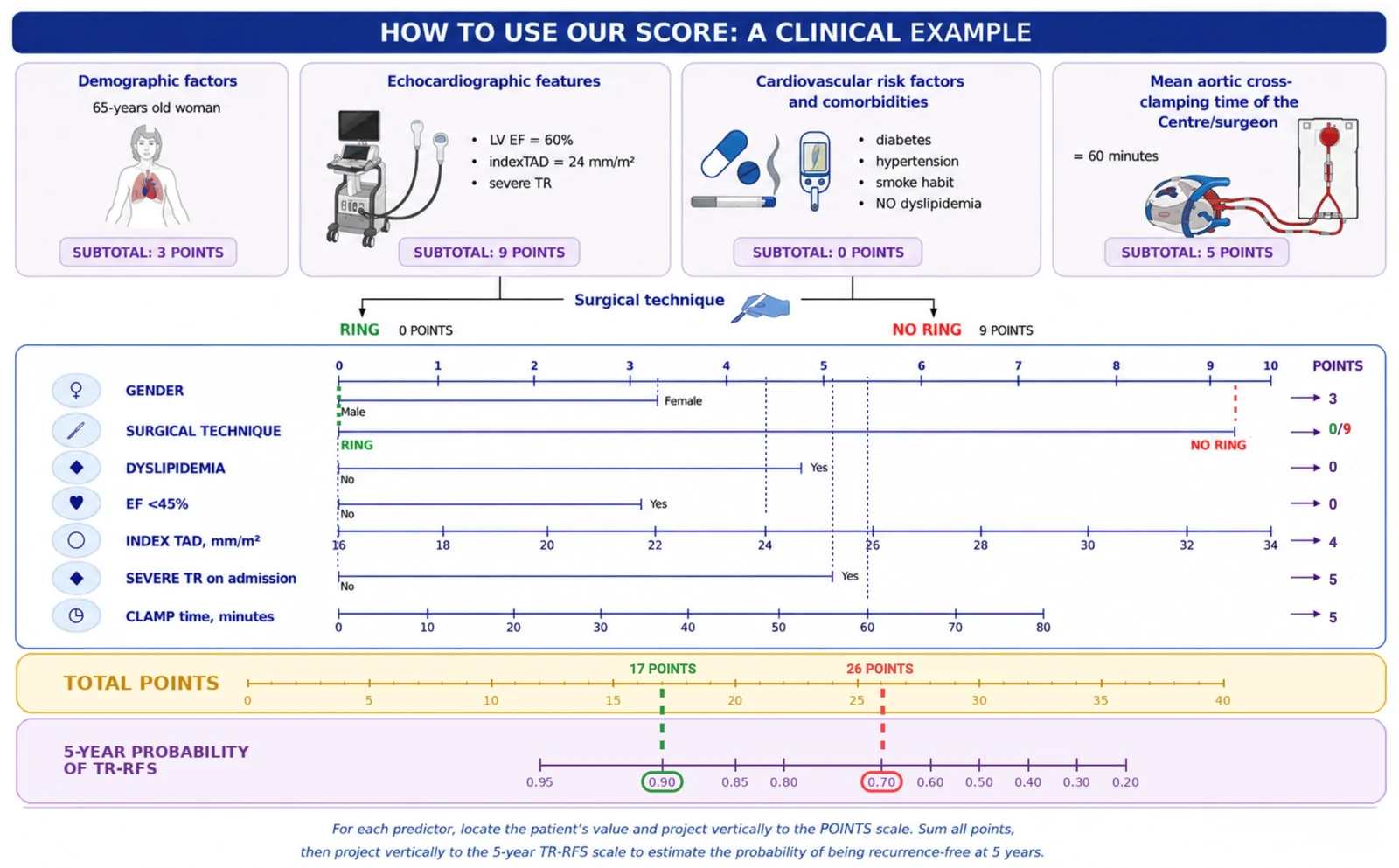
Example of individualized perioperative recurrence-risk estimation using the FuTURe nomogram. Each variable contributes proportionally to the overall score according to its regression coefficient. For operative variables, the aortic cross-clamp time should be interpreted as the average cross-clamp time routinely achieved by the operating surgeon or institution for combined mitral–tricuspid procedures, rather than the actual post-operative value of an individual patient. The selected surgical strategy is then incorporated into the model to estimate the corresponding 5-year probability of tricuspid regurgitation recurrence-free survival. This example illustrates the intended use of the nomogram as an exploratory perioperative decision-support tool within patients already selected for concomitant tricuspid repair (abbreviations: TR: Tricuspid Regurgitation; LV: Left Ventricle, EF: Ejection Fraction; TAD: Tricuspid Annulus Diameter; TR-RFS: Tricuspid Regurgitation Recurrence-Free Survival).

Based on their statistical significance, their clinical relevance, and the number of available outcome events according to the recommendations by Christodoulou et al. [23], these seven clinically relevant variables were entered into the multivariable Cox proportional hazards model. Surgical repair strategy emerged as the only independent predictor associated with recurrent TR, with suture-based annuloplasty showing a significantly higher recurrence risk than prosthetic ring annuloplasty (HR 3.75; *p* = 0.003). The model demonstrated satisfactory internal performance, with a C-index of 0.719 and good calibration following bootstrap internal validation (Figure 3). Regression coefficients were subsequently transformed into an exploratory individualized perioperative nomogram estimating the probability of 5-year recurrence-free survival (Figure 4). Figure 5 illustrates a practical example of individualized perioperative recurrence-risk estimation according to the surgical strategy under consideration.

## 4. Discussion

Despite increasing evidence supporting the treatment of functional tricuspid regurgitation (FTR), its surgical management remains inconsistent in contemporary clinical practice: the proportion of patients undergoing concomitant tricuspid valve (TV) repair during mitral valve surgery still varies markedly among institutions, ranging from less than 5% to more than 75% [24]. One important contributor to this variability is the growing adoption of minimally invasive mitral surgery, in which the additional complexity of concomitant tricuspid repair may discourage intervention. Consequently, despite progressively stronger guideline recommendations, undertreatment of FTR remains a significant unmet clinical need. Even among centers with high adherence to current guidelines, considerable variability persists in the choice of repair strategy. This real-world heterogeneity is reflected by the persistent recurrence of TR after mitral–tricuspid surgery, estimated at approximately 1.9% per year [8]. In this context, individualized estimation of recurrence risk may complement guideline-directed management by providing objective information to support surgical planning and long-term patient management. Accordingly, we investigated the principal clinical, echocardiographic, and surgical predictors of recurrent TR and developed the FuTURe nomogram, an individualized prediction model capable of estimating 5-year recurrence-free survival.

### 4.1. Clinical and Echocardiographic Factors

Our univariable analysis confirmed female sex as a predictor of recurrent TR, with women exhibiting approximately twice the risk of recurrence, in agreement with previous reports [25,26]. Considering the mean age of the female population included in our study, this finding may be partly explained by the greater tissue laxity associated with the postmenopausal period, potentially affecting valve integrity. Among echocardiographic variables, indexed tricuspid annular diameter and severe pre-operative TR were associated with recurrent disease. These findings are consistent with the concept proposed by Dreyfus et al., who suggested annular dilatation as the principal criterion for concomitant tricuspid intervention during mitral valve surgery [27]. Furthermore, left ventricular dysfunction (EF < 45%) was associated with a 3.26-fold increased risk of recurrence, corroborating previous observations [25,28]. A dilated and more spherical left ventricle may adversely affect right ventricular geometry and function through ventricular interdependence, thereby promoting recurrent TR [29].

### 4.2. Surgical Strategy and Individualized Risk Prediction

Prosthetic ring annuloplasty was independently associated with a lower probability of recurrent TR than suture-based annuloplasty in our cohort, consistent with previous studies demonstrating the superior long-term annular stabilization achieved by prosthetic rings [6,7,30,31,32]. Nevertheless, because repair strategy was not randomly allocated but reflected routine clinical decision-making, these findings should be interpreted as predictive associations rather than evidence of a causal treatment effect.

Current guidelines appropriately define when concomitant tricuspid repair should be performed and consistently support prosthetic ring annuloplasty whenever repair is indicated. However, surgical decision-making frequently extends beyond the simplified scenarios addressed by guidelines. Operative complexity, minimally invasive approaches, institutional philosophy, surgeon experience and patient-specific characteristics may still influence the choice of repair strategy in selected borderline situations. Although prosthetic ring annuloplasty represents the preferred contemporary technique, suture-based repairs continue to be employed in routine clinical practice because of their technical simplicity, shorter operative times, avoidance of prosthetic material and lower procedural complexity. In our institutional experience, the shorter cross-clamp times observed in the suture annuloplasty group were partly explained by the fact that selected ringless repairs were completed on the beating heart after aortic cross-clamp removal or during cardioplegia administration.

Rather than questioning current recommendations or suggesting equivalence between the two repair strategies, our findings acknowledge the persistence of this real-world variability and attempt to characterize the corresponding recurrence risk. In this context, the FuTURe nomogram is not intended to determine whether concomitant tricuspid repair should be performed, but rather to complement existing guideline recommendations by providing an individualized estimate of recurrence risk once the indication for repair has already been established. Although the present study was not designed to compare tricuspid repair with conservative management, individualized recurrence-risk assessment may contribute to narrowing the gap between evidence-based recommendations and everyday clinical practice by increasing surgeons’ confidence in tailoring the surgical strategy in carefully selected borderline scenarios. This potential implication should be regarded as hypothesis-generating and warrants prospective evaluation.

With the expanding role of transcatheter therapies for tricuspid valve disease, recent studies have increasingly emphasized the importance of optimizing the index operation to minimize the need for high-risk isolated surgical reintervention [33,34]. More broadly, the rapidly evolving landscape of structural heart interventions is progressively shifting surgical decision-making from an isolated procedure-oriented perspective toward a lifetime management strategy. As emphasized by Russo et al. [35], the characteristics of the index mitral valve intervention may substantially influence the feasibility, complexity and durability of subsequent transcatheter therapies. A similar concept may reasonably be extended to concomitant tricuspid surgery, where the initial repair strategy may influence future therapeutic options in patients who later develop recurrent TR. Consistent with this lifetime management perspective, prosthetic ring implantation may provide an additional long-term advantage by preserving the possibility of future valve-in-ring transcatheter intervention should recurrent TR occur [36]. Within this broader perspective, individualized estimation of recurrence risk should not be viewed merely as a prediction of procedural success, but rather as one component of personalized long-term management throughout the patient’s lifetime.

In this perspective, the nomogram should be regarded as an exploratory perioperative decision-support tool complementing existing guideline recommendations.

## 5. Conclusions

The FuTURe study improves the understanding of the determinants of recurrent functional tricuspid regurgitation following concomitant mitral–tricuspid surgery. Beyond identifying clinical, echocardiographic and operative predictors of recurrence, it proposes an exploratory individualized perioperative prediction model estimating long-term recurrence risk. Rather than challenging current guideline recommendations favoring prosthetic ring annuloplasty, the present study supports the broader concept that concomitant tricuspid surgery may benefit from a more personalized approach once the indication for repair has already been established. Within the evolving paradigm of lifetime structural heart disease management, individualized recurrence-risk estimation may represent one additional element supporting patient-centered surgical planning. External validation will be essential before routine clinical implementation. Consistent with its acronym, the FuTURe study encourages a forward-looking approach to functional tricuspid valve surgery by integrating evidence-based guideline recommendations with individualized recurrence-risk assessment, thereby promoting personalized surgical planning within a lifetime management perspective.

## 6. Study Limitations

Several limitations should be acknowledged. First, this was a single-center observational study, with retrospective collection of baseline and operative data and prospective follow-up. Second, the relatively limited number of recurrent TR events may have increased the risk of model overfitting despite restricting the number of candidate predictors and performing internal bootstrap validation. Third, treatment allocation was not randomized and reflected routine surgical decision-making; therefore, residual confounding cannot be excluded and the observed association between repair strategy and recurrent TR should be interpreted as predictive rather than causal. Fourth, recurrence was identified at scheduled echocardiographic examinations, introducing the possibility of interval censoring. Fifth, clinically established thresholds were adopted for selected continuous variables to improve clinical applicability of the nomogram, although modeling continuous predictors may provide greater statistical efficiency. Sixth, although De Vega and De Kay annuloplasty were analyzed as a single suture-based repair group because of their shared surgical concept and the limited sample size of each subgroup, subtle differences between the two techniques cannot be excluded and deserve evaluation in larger dedicated cohorts. Finally, although complete clinical follow-up was available for all included patients, vital status was ascertained through the institutional follow-up program rather than systematic linkage with regional or national mortality registries. Mortality findings should be interpreted within this context.

## Figures and Tables

**Table 1:** Demographic and clinical characteristics of the study sample on admission (n = 178).

	Overall (N = 178)	Ring (N = 54)	De Vega/De-Kay (N = 124)	
Demographic/ Anthropometric data				*p*
Age (yrs.), Mdn (IQR)	65 (57–73)	65 (59–72)	64.5 (56.0–73.0)	0.644
Gender, n (%)				0.103
Male sex, n (%)	87 (48.9)	21 (38.9)	66 (53.2)	
Female sex, n (%)	91 (51.1)	33 (61.1)	58 (46.8)	
BMI (kg/m^2^), Mdn (IQR)	24.7 (22.4–27.2)	24.9 (22.3–27.9)	24.6 (22.5–27.0)	0.324
Comorbidities				
Hypertension, n (%)	135 (75.8)	39 (72.2)	96 (77.4)	0.453
Type 2 diabetes mellitus, n (%)	16 (9.0)	7 (13.0)	9 (7.3)	0.257
Obesity, n (%)	15 (8.4)	7 (13.0)	8 (6.5)	0.156
Dyslipidemia, n (%)	72 (40.4)	22 (40.7)	50 (40.3)	1.000
Kidney failure, n (%)	19 (10.7)	8 (14.8)	11 (8.9)	0.291
COPD, n (%)	26 (14.6)	13 (24.1)	13 (10.5)	**0.022**
Neurological disorders, n (%)	11 (6.2)	5 (9.3)	6 (4.8)	0.312
Current smoking habit, n (%)	79 (44.4)	26 (48.1)	53 (42.7)	0.516
Peripheral edema, n (%)	7 (3.9)	5 (9.3)	2 (1.6)	**0.028**
Cardiovascular pre-operative data				
Atrial fibrillation, n (%)	79 (44.4)	32 (59.3)	47 (37.9)	**0.009**
Paroxysmal, n (%)	25 (14)	6 (11.1)	19 (15.3)	0.639
Persistent, n (%)	1 (0.5)	-	1 (0.8)	1.000
Permanent, n (%)	53 (29.8)	26 (48.1)	27 (21.8)	**<0.001**
NYHA Class				0.624
I-II, n (%)	82 (46.1)	23 (42.6)	59 (47.6)	
III-IV, n (%)	96 (53.9)	31 (57.4)	65 (52.4)	
Ejection Fraction (%), Mdn (IQR)	65 (58–71)	62.5 (55.0–69.0)	66.0 (59.7–71.0)	**0.018**
EF < 60%, n (%)	53 (29.8)	22 (40.7)	31 (25.0)	**0.049**
EF < 45%, n (%)	9 (5.1)	7 (13.0)	2 (1.6)	**0.004**
Anticoagulant therapy, n (%)	66 (37.1)	33 (61.1)	33 (26.6)	**<0.001**
Other pre-operative data				
PASP (mmHg), Mdn (IQR)	40 (30–50)	45.0 (40.0–58.7)	35.0 (30.0–45.2)	**<0.001**
PASP > 55 mmHg, n (%)	31 (17.4)	15 (27.8)	16 (12.9)	**0.030**
RA enlargement, n (%)				**<0.001**
Normal	48 (27.0)	7 (13.3)	41 (33.1)	
Mild	21 (11.8)	3 (5.6)	18 (14.5)	
Moderate	71 (39.9)	23 (42.6)	48 (38.7)	
Severe	38 (21.3)	21 (38.9)	17 (13.7)	
At least moderate RA enlargement, n (%)	109 (61.2)	44 (81.5)	65 (52.4)	**<0.001**
LAVI (mL/m^2^), Mdn (IQR)	72.4 (58.0–91.9)	82.6 (66.0–102.3)	68.7 (56.6–86.2)	0.006
LAVI > 60, n (%)	122 (68.5)	42 (77.8)	80 (64.5)	0.113
LVEDd (mm), Mdn (IQR)	34 (29–38)	33.0 (27.0–40.7)	34.0 (30.0–38.0)	0.919
LVEDd > 40 mm, n (%)	35 (19.7)	14 (25.9)	21 (16.9)	0.218
TAPSE (mm), Mdn (IQR)	24.0 (21.0–26.7)	22.0 (18.0–26.0)	24.7 (20.3–29.1)	**<0.001**
Severe TR, n (%)	38 (21.3)	21 (38.9)	17 (13.7)	**<0.001**
Index TAD (mm/m^2^), Mdn (IQR)	23.4 (2.8)	24.2 (2.8)	23.1 (2.7)	**0.024**
Index TAD > 21 mm/m^2^, n (%)	139 (78.1)	47 (87.0)	92 (74.2)	0.075
LV significant dilation, n (%)	58 (32.6)	22 (40.7)	36 (29.0)	0.164
Pre-operative RV dysfunction, n (%)	22 (12.4)	14 (25.9)	8 (6.5)	**0.001**

Abbreviations: BMI: Body Mass Index; COPD: Chronic Obstructive Pulmonary Disease; NYHA: New York Heart Association; EF: Ejection Fraction; PASP: Pulmonary Artery Systolic Pressure; RA: Right Atrial; LAVI: Left Atrial Volume Index; LVEDd: Left Ventricle End Diastolic Diameter; TAPSE: Tricuspid Annular Plane Systolic Excursion; TR: Tricuspid Regurgitation; TAD: Tricuspid Annulus Diameter; LV: Left Ventricle; RV: Right Ventricle. Bold values—*p* values ≤ 0.05.

**Table 2:** Surgical and follow-up data of the study sample (n = 178).

	Overall (N = 178)	Ring (N = 54)	De Vega/De-Kay (N = 124)	
Surgical Data				*p*
Surgical Technique				
Ring, n (%)	54 (30.3)	54 (100)	-	
De Vega/De-Kay, n (%)	124 (69.7)	-	124 (100)	
ECC (minutes), Mdn (IQR)	74.0 (59.3–102.7)	74.5 (61.0–101.4)	73.5 (56.0–98.0)	0.610
CLAMP (minutes), Mdn (IQR)	64.0 (55.0–83.6)	68.0 (55.2–85.5)	48.5 (45.7–78.2)	**0.011**
Concomitant procedures				
MVRepair, n (%)	126 (70.8)	44 (81.5)	82 (66.1)	**0.030**
MVReplacement, n (%)	52 (29.2)	10 (18.5)	42 (33.9)	**0.030**
Post-surgical data				
ICU LoS (days), Mdn (IQR)	2.0 (2.0–3.0)	2.5 (2.0–4.0)	2.0 (2.0–3.0)	0.055
Hospitalization LoS (days), Mdn (IQR)	6.5 (6.0–8.0)	7.0 (6.0–8.0)	6.0 (5.0–8.0)	0.057
In-hospital mortality, n (%)	-	-	-	n.a.
AVB, n (%)	43 (24.2)	13 (24.1)	30 (24.2)	1.000
PMK, n (%)	12 (6.7)	4 (7.4)	8 (6.5)	0.757
AF, n (%)	58 (32.6)	22 (40.7)	36 (29.0)	0.164
Follow-up data				
Mortality, n (%)	-	-	-	n.a.
PMK, n (%)	21 (11.8)	6 (11.1)	15 (12.1)	1.000
New cardiac surgery, n (%)	6 (3.4)	3 (5.5)	3 (2.4)	0.247
Re-hospitalization, n (%)	45 (25.3)	20 (37.0)	25 (20.2)	**0.024**
Cardiovascular re-hospitalization, n (%)	23 (12.9)	11 (20.4)	12 (9.7)	0.086
Outcome				
Recurrent TR, n (%)	38 (21.3)	8 (14.8)	30 (24.2)	**0.014**
Time to recurrent TR (months), Mdn (IQR)	69.7 (48.1–85.8)	85.6 (58.6–99.0)	56.3 (46.3–70.5)	**<0.001**

Abbreviations: ECC: Extra-Corporeal Circulation; MV: mitral valve; ICU: Intensive Care Unit; LoS: Length of Stay; AVB: atrio-ventricular block; PMK: pace-maker; AF: atrial fibrillation; TR: tricuspid regurgitation. Bold values—*p* values ≤ 0.05.

**Table 3:** Predictors of recurrent tricuspid regurgitation (N = 178).

	Recurrent TR	No Recurrent TR	Ordinary Cox Model	Multivariable Analysis
	N = 38	N = 140	HR (95%CI); *p*	HR (95%CI); *p*
Demographic and anthropometric data				
Age (yrs.), Mdn (IQR)	68.5 (63.2–74.5)	64.5 (55.7–73.0)	1.04 (1.00; 1.07); 0.060	
Female sex, n (%)	28 (73.7)	63 (45.0)	**2.09 (1.01; 4.32); 0.045**	1.55 (0.70; 3.44); 0.281
BMI (kg/m^2^), Mdn (IQR)	25.0 (4.3)	25.0 (3.5)	1.00 (0.91; 1.10); 0.992	
Comorbidities				
Hypertension, n (%)	29 (76.3)	106 (75.7)	1.37 (0.65; 2.92); 0.408	
Type 2 diabetes mellitus, n (%)	4 (10.5)	12 (8.6)	1.54 (0.54; 4.38); 0.415	
Obesity, n (%)	3 (7.9)	12 (8.6)	1.16 (0.35; 3.83); 0.801	
Dyslipidemia, n (%)	19 (50.0)	53 (37.9)	**1.95 (1.00; 3.83); 0.050**	1.93 (0.96; 3.91); 0.066
Kidney failure, n (%)	3 (7.9)	16 (11.4)	1.49 (0.56; 3.91); 0.422	
COPD, n (%)	4 (10.5)	22 (15.7)	0.86 (0.30; 2.44); 0.771	
Neurological disorders, n (%)	3 (7.9)	8 (5.7)	0.75 (0.23; 2.47); 0.642	
Current smoking habit, n (%)	15 (39.5)	64 (45.7)	0.98 (0.50; 1.89); 0.944	
Peripheral edema, n (%)	1 (2.6)	6 (4.3)	1.71 (0.42; 6.93); 0.449	
Previous anticoagulant therapy, n (%)	19 (50)	47 (33.6)	1.08 (0.56; 2.10); 0.817	
Cardiovascular pre-operative data				
Atrial fibrillation, n (%)	23 (60.5)	56 (40.0)	1.31 (0.66; 2.61); 0.432	
NYHA class				
I-II, n (%)	14 (36.8)	68 (48.6)	-	
III-IV, n (%)	24 (63.2)	71 (51.4)	1.35 (0.69; 2.64); 0.379	
Ejection fraction (%), Mdn (IQR)	65.0 (58.5–72.7)	65.0 (58.0–70.0)	1.02 (0.99; 1.06); 0.199	
EF < 60%, n (%)	10 (26.3)	43 (30.7)	0.65 (0.31; 1.35); 0.247	
EF < 45%, n (%)	3 (7.9)	6 (4.3)	**3.26 (1.56; 6.84); 0.002**	1.57 (0.42; 5.83); 0.497
PASP (mmHg), Mdn (IQR)	45 (35–50)	40 (30–50)	1.00 (0.98; 1.02); 0.888	
PASP > 55 mmHg, n (%)	8 (21.1)	23 (16.4)	1.06 (0.48; 2.32); 0.887	
Moderate RA enlargement, n (%)	25 (65.8)	84 (60.0)	0.92 (0.47; 1.81); 0.817	
LAVI (mL/m^2^), Mdn (IQR)	83.7 (62.4–112.6)	71.4 (57.9–88.2)	1.00 (1.00; 1.01); 0.701	
LAVI > 60 mL/m^2^, n (%)	28 (73.7)	94 (67.1)	0.97 (0.46; 2.02); 0.935	
LVEDd (mm), Mdn (IQR)	31.0 (27.2–37.5)	34.0 (30.0–38.0)	0.99 (0.95; 1.03); 0.519	
LVEDd > 40 mm, n (%)	9 (23.7)	26 (18.6)	1.05 (0.49; 2.23); 0.899	
TAPSE (mm), Mdn (IQR)	23.4 (18.6–28.2)	24.0 (19.7–28.3)	1.00 (0.91; 1.09); 0.984	
Index TAD (mm), Mdn (IQR)	24.3 (21.6–27.0)	23.2 (20.4–26.0)	**1.13 (1.01; 1.27); 0.033**	1.08 (0.94; 1.24); 0.263
Index TAD > 21 mm, n (%)	34 (89.5)	105 (75.0)	2.15 (0.76; 6.08); 0.148	
Severe TR, n (%)	14 (36.8)	24 (17.1)	1.77 (0.91; 3.45); 0.095	2.05 (0.94; 4.46); 0.070
LV significant dilation, n (%)	13 (34.2)	45 (32.1)	1.65 (0.82; 3.31); 0.158	
Pre-operative RV dysfunction, n (%)	5 (13.2)	17 (12.1)	1.36 (0.53; 3.50); 0.528	
Surgical data				
Surgical technique				
De Vega/De-Kay, n (%)	30 (78.9)	94 (67.1)	**2.62 (1.18; 5.81); 0.018**	**3.75 (1.58; 8.93); 0.003**
Ring, n (%)	8 (21.1)	46 (32.9)	**-**	
CLAMP time (minutes), Mdn (IQR)	83.5 (56.2–100.7)	68.5 (49.0–88.0)	**1.02 (1.01; 1.03); 0.004**	1.01 (1.00; 1.03); 0.077

Abbreviations: TR: Tricuspid Regurgitation; BMI: Body Mass Index; COPD: Chronic Obstructive Pulmonary Disease; NYHA: New York Heart Association; EF: Ejection Fraction; PASP: Pulmonary Artery Systolic Pressure; RA: Right Atrial; LAVI: Left Atrial Volume Index; LVEDd: Left Ventricle End Diastolic diameter; TAPSE: Tricuspid Annular Plane Systolic Excursion; TAD: Tricuspid Annulus Diameter; TR: Tricuspid Regurgitation; LV: Left Ventricle; RV: Right Ventricle; HR: Hazard Ratio; CI: Confidence Interval. Bold values—*p* values ≤ 0.05.

## Data Availability

The data presented in this study are available on request from the corresponding author. The data are not publicly available due to privacy and ethical restrictions regarding patient confidentiality.

## Abbreviations

The following abbreviations are used in this manuscript:

TR	Tricuspid Regurgitation
FTR	Functional Tricuspid Regurgitation
TV	Tricuspid Valve
LV	Left Ventricle
EF	Ejection Fraction
RV	Right Ventricle
RFS	Recurrence-Free Survival
TAD	Tricuspid Annulus Diameter
